# Hermansky-Pudlak Syndrome Type 2: A Case Report on an Ultra-Rare Disorder

**DOI:** 10.7759/cureus.65114

**Published:** 2024-07-22

**Authors:** Badriah G Alasmari, Shady Wafa, Ali M Tahir, Abdullah Aljubran, Adel Alfaifi, Khulod Alsaab, Lina Elzubair

**Affiliations:** 1 Pediatrics, Armed Forces Hospital Southern Region, Khamis Mushait, SAU; 2 Dermatology, Armed Forces Hospital Southern Region, Khamis Mushait, SAU; 3 Hematopathology, Armed Forces Hospital Southern Region, Khamis Mushait, SAU

**Keywords:** neutrophil count, epistaxis, skin lesions, oculocutaneous albinism (oca), hermansky-pudlak syndrome type 2

## Abstract

Hermansky-Pudlak syndrome (HPS) is an infrequent entity, with a multisystem involvement and autosomal recessive inheritance involving genetic mutations that lead to defective organelles of lysosomes. HPS is characterized by oculocutaneous albinism, platelet storage deficiency associated with prolonged bleeding, pulmonary fibrosis, and granulomatous colitis.

In our case report, we describe a two-year-old boy with the clinical presentation of oculocutaneous albinism, generalized skin lesions, and recurrent bilateral epistaxis since the age of one year. He was diagnosed with HPS type 2 based on the clinical findings and supported by a genetic study that confirmed the loss of exon 23-24 of the *AP3B1* gene.

## Introduction

Hermansky-Pudlak (HPS) syndrome is an infrequent disease in clinical practice with a multisystem involvement. It was first elaborated in 1959. More than half of reported cases belong to Puerto Rico with a published frequency of 1:1800. This syndrome is delineated with oculocutaneous albinism, a bleeding diathesis due to platelet storage deficiency, and other manifestations like neutropenia, pulmonary fibrosis, or granulomatous colitis [1]. Different gene mutations are inferred in 11 separate forms of HPS. It is characterized by defects in the trafficking of intracellular protein and the functioning of lysosome-related organelles like melanosomes or platelet-dense granules [2]. The clinical manifestations do vary among affected individuals. Hermansky-Pudlak type 1 (HPS1) is the most common subtype and encompasses a debilitating progressive pulmonary fibrosis, impairing the quality of life [3]. The HPS2 subtype is quite rare as less than 40 cases are reported worldwide. Neutropenia is one of the major distinguishing features of HPS2, as it confers from mutations in the AP3B1 gene (autosomal recessive inheritance). Neutropenia predisposes toward recurrent pulmonary infection, however, it improves with granulocyte colony-stimulating factor. The pulmonary disease entails altered alveolar epithelial type II cells, which leads to defective intracellular processing of surfactant proteins B and C, premature apoptosis, and fibrosis. Pulmonary fibrosis has not been reported in HPS3 and HPS5 through HPS10 [4].

Here, we report a new case of a toddler male from the southern region of Saudi Arabia who presented with recurrent epistaxis and generalized skin lesions with chronic neutropenia.

## Case presentation

We present a case of a two-year-old male child delivered to second-degree consanguineous parents. He was a full-term baby born via normal vaginal delivery without neonatal intensive care unit (NICU) admission. He was the first baby with a younger sibling alive and healthy. Moreover, he had a significant family history, as his cousin had oculocutaneous albinism but without any confirmed diagnosis.

He had fair skin since birth. Till the age of nine months, he had an indolent course when he started to have recurrent fever with skin lesions, which became vesicles and then crusted; this was noted to recur three times a year. Moreover, he was found to have recurrent bilateral epistaxis with chronic cough. He underwent tonsillectomy at the age of 16 months due to recurrent tonsillitis with a benign postoperative course with no considerable bleeding. At the age of two years, the patient was referred to the pediatric hematology clinic from the Ear, Nose, Throat (ENT) clinic with concerns for episodic bilateral epistaxis during the last year. There was no history of bleeding from any other orifice or delayed umbilical cord separation. He had no joints or bone pain.

**Figure 1:**
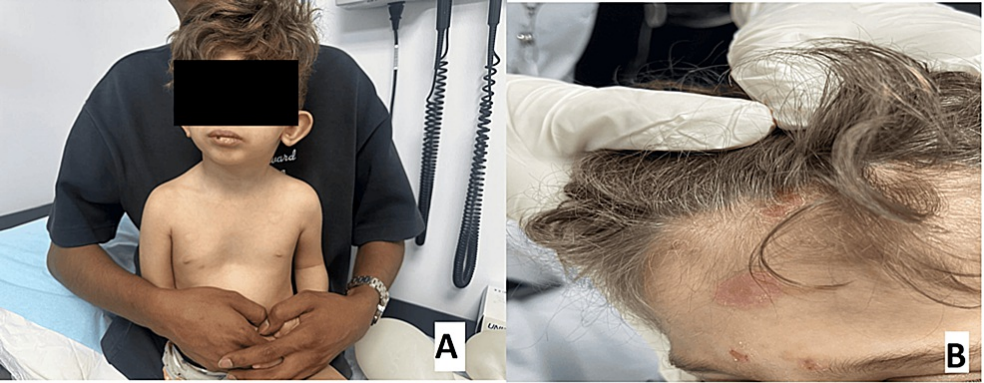
(A) Generalized hypopigmentation of the skin compared to his parents; (B) light-colored hair and multiple erosions over the face and scalp

Physical examination revealed dysmorphic features in the form of abnormal light coloring of the skin, hair, and eyes with low-set and posteriorly rotated ears, broad nasal root, retrognathia, and thin upper lip. Skin examination revealed generalized hypopigmentation of the skin (types I-II) compared to his parents (type IV-V), light-colored hair (Figure 1A), multiple erosions over the face, scalp, and axillae, with some of them having honey-colored crusts, scattered erythematous, tender skin nodules (Figure 1B).

Other systemic examination was normal with growth parameters in the normal range. Our patient was admitted from the clinic due to severe and chronic neutropenia for more evaluation and treatment (Table 1). The patient received antibiotics for skin infection and granulocyte colony-stimulating factor (GCSF) for five days, without any response.

**Table 1:** Laboratory workup exhibiting neutropenia and platelet dysfunction TWBC = total white blood cell count, INR = international normalized ratio, APTT = activated partial thromboplastin clotting time, EPI = epinephrine, ADP = adenosine diphosphate

Laboratory parameters	Results	Units	Reference Range
TWBC	7.27	x 10^9^/L	4.5-13.5
RBC	3.59	x 10^12^/L	4.1-5.3
Hb	8.23	g/dL	10.9-15
MCV	73.65	fL	73-89
MCH	22.93	Pg	23-30
Platelets	618	x 10^9^/L	150-450
Neutrophils absolute	0.27	x 10^9^/L	1.5-8.5
Lymphocytes absolute	6.08	x 10^9^/L	2-8
Platelet Function Assay			
Collagen/EPI	248	Sec	85-165
Collagen/ADP	125	Sec	71-118
INR	1.19		<1.2
PT	16.40	Sec	11 -13.5
APTT	27.40	Sec	26-40

**Figure 2:**
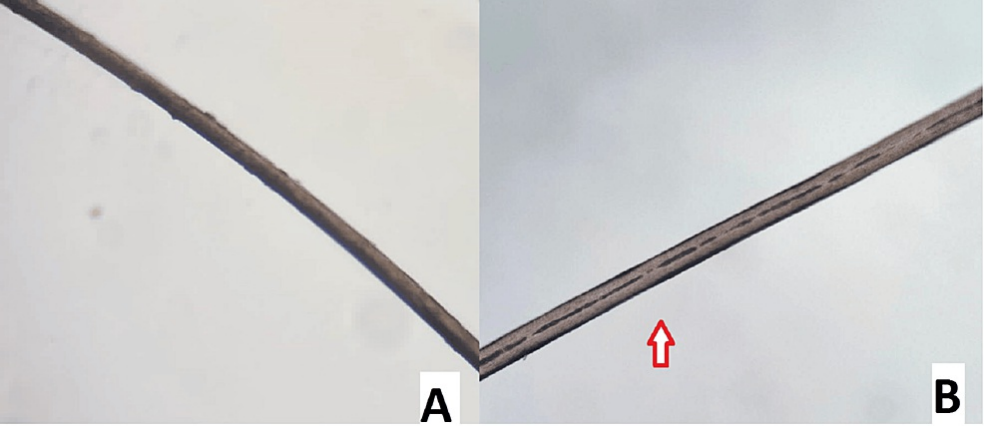
Microscopic hair shaft examination showed reduced hair shaft pigmentation (red arrow) in the patient (B) as compared to the control (A)

Microscopic hair shaft examination showed reduced hair shaft pigmentation in the patient compared to the control, but no abnormal clumping of pigment was seen (Figure 2).

**Figure 3:**
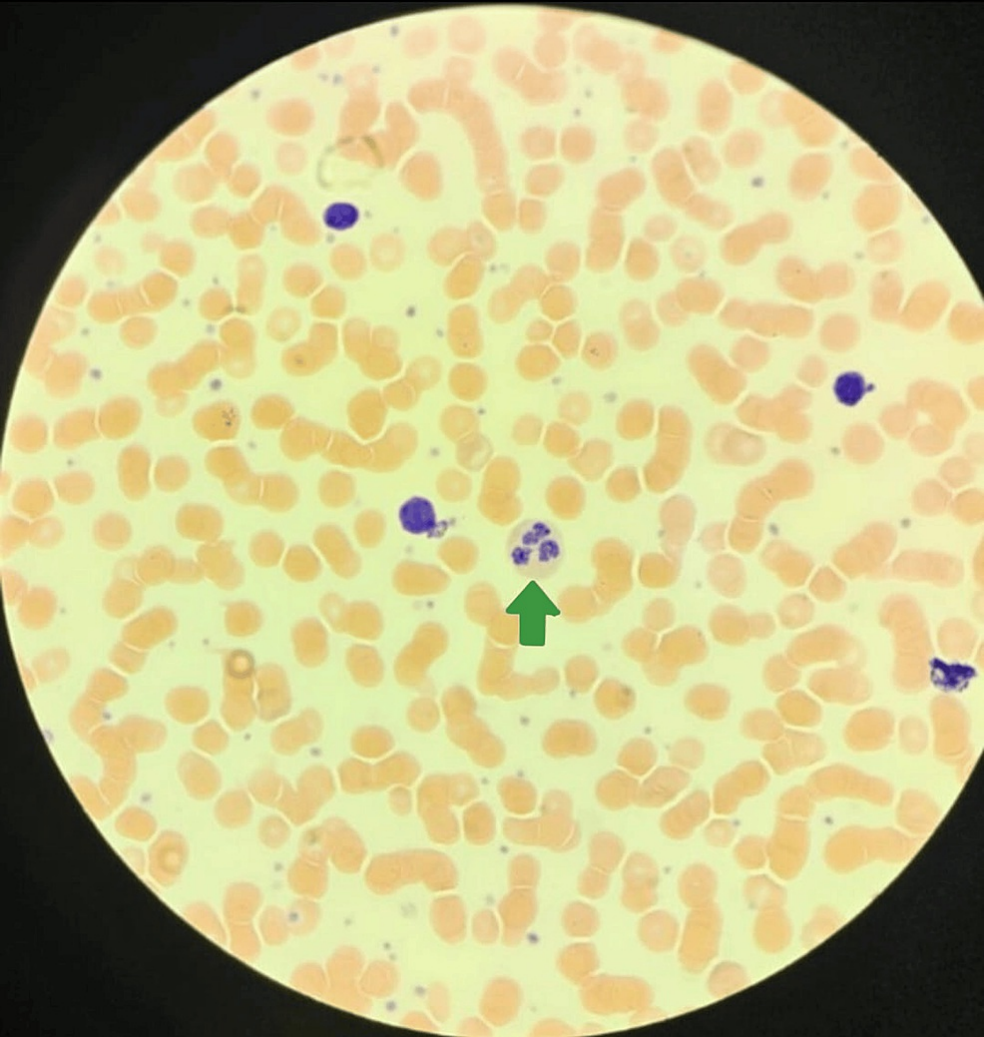
Peripheral blood smear with Wright-Giemsa stain showing severe neutropenia with some dysplastic nuclear segmentation and hypogranulation (green arrow)

Peripheral blood smear showed hypochromic microcytic RBC with occasional polychromasia (suggesting bleeding on top of iron deficiency anemia), rouleaux formation, and anisopoikilocytosis +1. There was severe neutropenia with some dysplastic nuclear segmentation and hypogranulation, occasional activated lymphocytes with no blasts, along with mild thrombocytosis with normal-sized platelets and markedly prolonged closure times indicating platelet dysfunction (Figure 3).

Skin culture from wound ulcer showed *Staphylococcus aureus*. All radiological examinations were normal. Hearing assessment, ophthalmological examination, and screening for other congenital malformations turned out to be normal. He is on follow-up with the hematology clinic with persistent neutropenia and an improved rash. A genetic WES (whole exome sequencing) study result showed autosomal recessive Hermansky-Pudlak syndrome type 2 involving exon 23-24 of the *AP3B1* gene.

## Discussion

Hermansky-Pudlak described HPS in 1959. They exhibited two cases with identical symptoms of albinism, bleeding tendency, bizarre pigmented reticular cells in the bone marrow, and lung involvement [5]. In terms of confirming the diagnosis, ocular albinism along with evidence of lack of platelet storage capacity is required. Genetic study is essential for elaborating the type of HPS and thus judging the prognosis. The classic picture embarks on albinism with bleeding tendency, colitis, and pulmonary fibrosis [6].

We reported a case of HPS2 who presented with skin hypopigmentation, bleeding tendency, and clinical immunodeficiency phenotype, characterized by persistent neutropenia and recurrent infection. In our case, phenotype strongly suggested HPS2, so we confirmed the diagnosis with the WES study, as it resembles other secretopathies like Chediak Higashi syndrome, Griscelli syndrome, and familial hemophagocytic lymphohistiocytosis (HLH) disorders, which might become complicated by episodes of the hemophagocytic syndrome in response to viral infection.

A study was published mentioning six children with genetically proven HPS2 and documented to the chILD-EU register between 2009 and 2017. It emerged from epistaxis during the initial two years of age. Insidious but trivial respiratory symptoms led to delayed diagnosis at the age of five years [7].

In another report, a five-month-old female infant presented with respiratory sepsis requiring ventilation, and clinical examination revealed occulocutaneous albinism and hepatosplenomegaly. Laboratory data revealed severe neutropenia, CD4 lymphopenia, normal immunoglobulins, marked reduction of platelet nucleotides, and abnormal platelet aggregation studies [8].

A case scenario had reported granulomatous colitis associated with HPS. Twenty percent of patients with HPS are associated with granulomatous colitis in the first or third decades of life, especially types 1 and 4, so less likely to be associated with HPS2 like our patient presentation [5].

Compared with other studies and reports about HPS2, our patient has oculocutaneous albinism, a bleeding diathesis, and neutropenia but no pulmonology, gastroenterology, or other ophthalmological manifestations.

Treatment of Heřmanský-Pudlák syndrome consists of multidisciplinary specialties. Recognizing clinical symptoms with prompt diagnosis and preventing complications is the cornerstone of management.

This case report serves as a momentous scientific assistance by elucidating the occurrence of a rare entity of HPS2. It brings to the limelight the pivotal role of timely appreciation of this rare association, potentially steering toward appropriate interventions and improved clinical outcomes. Moreover, it advocates the use of a multidisciplinary approach while intercepting such intricate clinical challenges.

## Conclusions

Hermansky-Pudlak syndrome (HPS) should be considered as the differential diagnosis in patients presenting with oculocutaneous albinism, recurrent infection, bleeding diathesis, visual problems, or dyspnea. We suggest a genetic WES study in strongly suspected cases to differentiate other similar conditions like Chediak Higashi syndrome, Griscelli syndrome, and familial hemophagocytic lymphohistiocytosis (HLH) disorders. We present this case with a typical clinical picture of Hermansky Pudlak syndrome type 2 (HPS2) so physicians are more aware of the disease and early diagnosis can aid in a favorable prognosis.

## References

[1] Vicary GW, Vergne Y, Santiago-Cornier A, Young LR, Roman J (2016). Pulmonary fibrosis in Hermansky-Pudlak syndrome. Ann Am Thorac Soc.

[2] Gochuico BR, Huizing M, Golas GA (2012). Interstitial lung disease and pulmonary fibrosis in Hermansky-Pudlak syndrome type 2, an adaptor protein-3 complex disease. Mol Med.

[3] Jessen B, Bode SF, Ammann S (2013). The risk of hemophagocytic lymphohistiocytosis in Hermansky-Pudlak syndrome type 2. Blood.

[4] Schinella RA, Greco MA, Cobert BL, Denmark LW, Cox RP (1980). Hermansky-Pudlak syndrome with granulomatous colitis. Ann Intern Med.

[5] Hussain N, Quezado M, Huizing M, Geho D, White JG, Gahl W, Mannon P (2006). Intestinal disease in Hermansky-Pudlak syndrome: occurrence of colitis and relation to genotype. Clin Gastroenterol Hepatol.

[6] Sandrock-Lang K, Bartsch I, Buechele N, Koehler U, Simon-Gabriel CP, Eckenweiler M, Zieger B (2017). Novel mutation in two brothers with Hermansky Pudlak syndrome type 3. Blood Cells Mol Dis.

[7] Hengst M, Naehrlich L, Mahavadi P (2018). Hermansky-Pudlak syndrome type 2 manifests with fibrosing lung disease early in childhood. Orphanet J Rare Dis.

[8] Sofia MA, Sakuraba A, Rubin DT (2017). Two complex cases of Hermansky-Pudlak syndrome highlight a potential biologic explanation for an associated Crohn’s disease phenotype. ACG Case Rep J.

